# Single-cell dissection of PTM-related networks reveals an immunosuppressed osteosarcoma ecosystem

**DOI:** 10.3389/fmolb.2025.1718941

**Published:** 2025-12-18

**Authors:** Jingyu Chen, Wei Zhang, Hai Yan, Jinyu Chen, Hanrui Liu, Xingyu Zhou, Haiping Zhang, Dongdong Cheng

**Affiliations:** The Second Affiliated Hospital of Nantong University, Nantong, China

**Keywords:** osteosarcoma, post-translational modification, single-cell RNA sequencing, tumor microenvironment, prognostic signature, GRN

## Abstract

**Background:**

Osteosarcoma remains lethal for many patients with metastatic or relapsed disease. Post-translational modifications (PTMs) regulate protein signaling and may shape the tumor microenvironment and clinical behavior in osteosarcoma, but PTM-anchored transcriptomic programs are as yet not well defined.

**Methods:**

We integrated single-cell RNA sequencing from GSE162454 with curated PTM and immune gene sets to build a PTM-related framework for osteosarcoma. Tumor cell differentially expressed genes were intersected with PTM and immune repertoires to derive candidates. A PTM-related prognostic score was trained in TARGET-OS and validated in GSE21257 and GSE16091. Immune infiltration and microenvironment features were profiled using ssGSEA, Estimation of STromal and Immune cells in MAlignant Tumor tissues using Expression (ESTIMATE) data, and Tumor Immune Dysfunction and Exclusion (TIDE) scores. Model interpretation used SHapley Additive exPlanations (SHAP) and single-cell localization. GRN was prioritized for exploration of immune correlations and *in vitro* loss-of-function assays in U2OS and HOS cells.

**Results:**

The three-way intersection yielded 298 genes. The PTM-related score stratified overall survival in training and validation cohorts and remained independent of clinical covariates. High scores aligned with an immunosuppressed, stroma-rich microenvironment, with lower ImmuneScores and ESTIMATE scores, enrichment of myeloid and regulatory lineages, higher dysfunction and exclusion by TIDE, and reduced cytolytic, interferon, and antigen-presentation programs. SHAP highlighted a compact driver set enriched in malignant and stromal compartments. GRN showed strong contribution and consistent single-cell localization. Elevated GRN correlated with plasmacytoid dendritic cells, myeloid-derived suppressor cells (MDSCs), macrophages, regulatory T cells (Tregs), and multiple inhibitory checkpoints and with diminished immune effector functions. GRN silencing reduced proliferation, clonogenicity, migration, and invasion in osteosarcoma cells.

**Conclusion:**

A PTM-anchored transcriptomic signature captures prognostic heterogeneity in osteosarcoma and links adverse outcome to an immunosuppressed microenvironment. GRN emerges as a tumor- and stroma-intrinsic mediator of immune suppression and malignant traits and represents a biologically grounded target for future mechanistic and therapeutic studies.

## Introduction

Osteosarcoma (OS) is the most common primary malignant bone tumor of childhood and adolescence, characterized by aggressive local invasion, early metastatic spread, and substantial molecular heterogeneity (Kansara et al., 2014; Chen Y. et al., 2021). Standard multimodal therapy has improved outcomes for some patients, yet durable control remains challenging—particularly in metastatic or relapsed settings—underscoring the need for biologically grounded biomarkers and therapeutic targets (Khadembaschi et al., 2022). Increasing evidence suggests that the tumor microenvironment (TME), including stromal and immune compartments, plays a decisive role in OS progression and treatment response, but the molecular programs that orchestrate these interactions are incompletely understood (Chen C. et al., 2021; Anwar et al., 2020). Recent syntheses emphasize that OS harbors a profoundly myeloid-dominant, T-cell-excluded ecosystem, with heterogeneous macrophage, dendritic, and fibroblastic programs that shape immune evasion and therapy resistance. Single-cell and spatial studies delineate suppressive dendritic subsets, diversified myeloid states, and stromal-immune crosstalk that collectively blunt cytotoxic T-cell activity and impede checkpoint efficacy in OS (Tian et al., 2023).

Post-translational modifications (PTMs), such as phosphorylation, acetylation, ubiquitination, glycosylation, and proteolytic processing, govern protein stability, localization, and signaling dynamics (Vu et al., 2018; Patwardhan et al., 2021). Aberrant PTM wiring is a hallmark of cancer and can reprogram lineage differentiation, extracellular matrix remodeling, stress responses, and immune recognition (Pan and Chen, 2022; Chen et al., 2020). In bone malignancies, PTM enzymes and substrates have been linked to osteogenic lineage control, metastatic competence, and drug resistance (Kong et al., 2025; Mao et al., 2024). However, a systematic, transcriptome-wide delineation of PTM-related gene programs in OS—particularly in relation to the immune milieu and at single-cell resolution—remains lacking. PTMs critically regulate tumor–immune interactions by controlling checkpoint stability, receptor trafficking, antigen presentation, and cytokine signaling, thereby shaping immune recognition and responsiveness to immune checkpoint blockade (ICB) across cancers (Jia et al., 2025).

Single-cell RNA sequencing (scRNA-seq) enables unbiased deconvolution of malignant, stromal, and immune populations in osteosarcoma, and curated PTM and immune gene sets anchor pathway-level inferences to mechanistic biology (Zheng et al., 2024; Zhou et al., 2020). By integrating these resources, we can delineate PTM-anchored transcriptional features within the OS ecosystem, connect PTM activity to patterns of immune infiltration, dysfunction, and exclusion, and derive clinically meaningful signatures that support risk stratification and therapeutic decision-making. Recent multi-omics studies in solid tumors have shown that immune microenvironment patterns strongly influence prognosis and therapeutic response and that integrative signatures can effectively stratify patients according to their immune landscapes (Zhang et al., 2023).

Accordingly, this study leverages scRNA-seq and bulk transcriptomic cohorts to map PTM-related gene networks in OS, examine their associations with the TME and antitumor immunity, and explore therapeutic implications, including potential molecular targets and drug sensitivity relationships. By focusing on PTM biology at the cellular and pathway levels, we aim to provide a framework that links fundamental protein regulation to clinically relevant phenotypes in OS.

## Methods

### Data sources and cohorts

Single-cell RNA-seq data were obtained from GSE162454 (Liu et al., 2024). Bulk transcriptomic and clinical data were collected from TARGET-OS (training cohort) and two external Gene Expression Omnibus (GEO) validation cohorts (GSE21257 (Buddingh et al., 2011) and GSE16091 (Paoloni et al., 2009)). For immunotherapy analyses, a urothelial carcinoma anti-PD-L1 cohort (IMvigor210) with response annotations was used as a pharmacodynamic surrogate. All datasets were de-identified and accessed in accordance with their respective data-use terms. Nine PTM categories were assembled from curated resources and literature and merged into a non-redundant PTM gene universe. Immune-related gene sets were compiled from hallmark and immune gene set catalogs.

### Construction of the PTM-related prognostic score (PTMS)

In TARGET-OS, univariable Cox regression was applied to the core candidates to pre-select survival-associated genes (P < 0.05). A LASSO-Cox model then identified a parsimonious gene panel. The PTMS was computed as a weighted sum of expression values. Patients were dichotomized at the median PTMS level unless otherwise specified.

### Immune infiltration and tumor microenvironment scoring

Bulk immune infiltration was estimated with ssGSEA/GSVA against curated immune cell signatures. Estimation of STromal and Immune cells in MAlignant Tumor tissues using Expression (ESTIMATE) data provided ImmuneScore, StromalScore, and ESTIMATE score values. A composite TMEscore-A/B and a combined TMEscore were calculated following published definitions. Tumor Immune Dysfunction and Exclusion (TIDE) scores and components (dysfunction, exclusion) were computed. Immune checkpoint/receptor–ligand expression and immune function modules were compared across PTMS or GRN-based strata.

### Model interpretation

Feature contributions to PTMS were quantified using SHapley Additive exPlanation (SHAP) values (for Cox models), generating importance rankings and beeswarm dependence plots in the training and validation cohorts.

### Drug–response prediction

Chemotherapy sensitivity was inferred using transcriptome-based IC_50_ prediction trained on pharmacogenomic references, yielding per-sample predicted IC_50_ values for paclitaxel, docetaxel, bortezomib, gemcitabine, cisplatin, and methotrexate. Group comparisons employed Wilcoxon rank-sum tests with the Benjamini–Hochberg (BH) correction.

### Statistics and reproducibility

Continuous variables were compared using the Wilcoxon or t-test, as appropriate. Correlations used Spearman’s method. Multiple testing was controlled using the Benjamini–Hochberg procedure (false discovery rate (FDR) q < 0.05). All tests were two-sided. The overall analytical workflow is illustrated in Supplementary Figure S1.

## Results

### Single-cell atlas and intersection of PTM/immune-related differentially expressed genes (DEGs)

**FIGURE 1:**
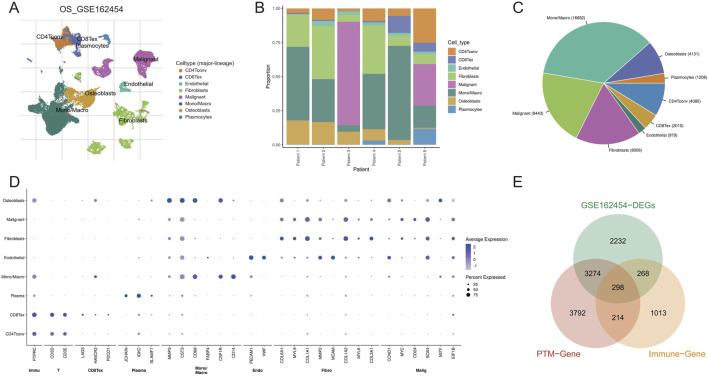
Single-cell atlas of OS and intersection of tumor differentially expressed genes (DEGs) with PTM/immune genes. **(A)** UMAP of GSE162454 showing annotated major lineages. **(B)** Cell-type composition per patient. **(C)** Overall cell-number proportion across the cohort. **(D)** Dot plot of canonical markers validating the annotations. **(E)** Venn diagram of tumor cell DEGs overlapped with PTM- and immune-related gene sets, yielding a three-way intersection of 298 genes.

Using the GSE162454 tumor samples, we performed dimensionality reduction and cell-type annotation, identifying eight major lineages—malignant cells, monocytes/macrophages (Mono/Macro), fibroblasts, osteoblasts, endothelial cells, CD4 Tconv lymphocytes, CD8 Tex cells, and plasma cells (Figure 1A). Cell-type composition varied across patients, with Mono/Macro and malignant cells predominating (Figures 1B,C). Canonical markers supported the accuracy of these annotations (Figure 1D). Intersecting tumor cell DEGs with nine PTM gene sets and immune-related genes yielded 298 core candidate genes (Figure 1E).

### Construction and validation of a PTM-related prognostic signature

**FIGURE 2:**
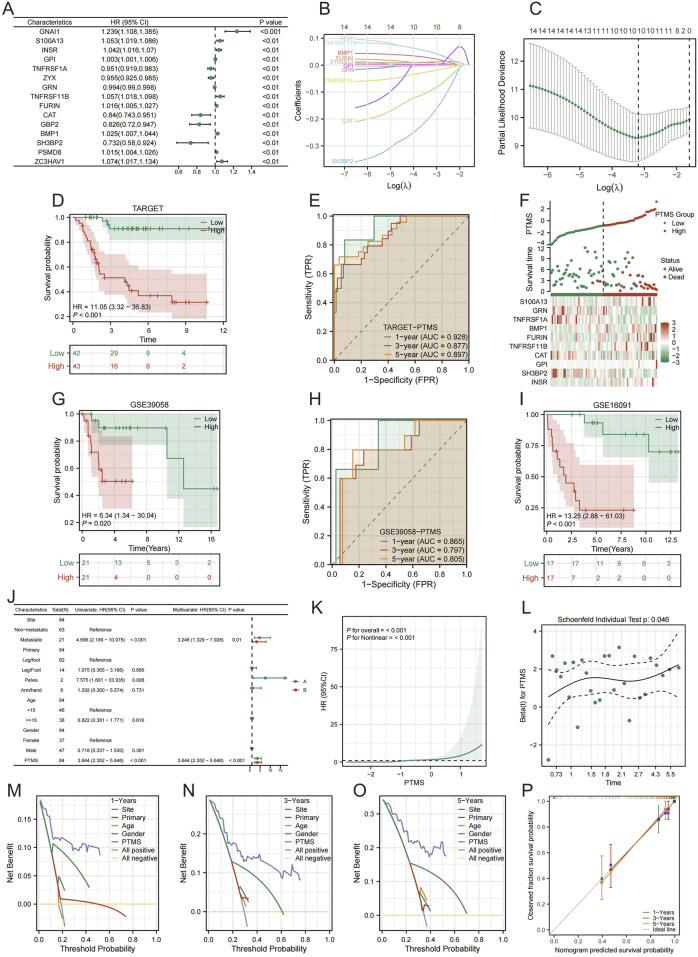
Development and validation of a PTM-related prognostic score. **(A)** Univariable Cox forest plot of candidate genes in TARGET. **(B,C)** LASSO-Cox modeling: coefficient paths and 10-fold CV to select λ. **(D,E)** TARGET training set: KM overall survival by PTMS **(D)** and time-dependent ROC **(E)**. **(F)** Risk stratification in TARGET: PTMS distribution, survival status, and model-gene heatmap. **(G,H)** GSE21257 validation: KM **(G)** and ROC **(H)**. **(I)** GSE16091 validation: KM. **(J)** Multivariable Cox confirms PTMS as an independent factor. **(K)** Restricted cubic spline showing increasing hazard with higher PTMS. **(L)** Proportional-hazards diagnostics of PTMS. **(M–O)** Decision-curve analysis at 1, 3, and 5 years of PTMS. **(P)** Calibration of predicted vs. observed survival.

From the 298 core candidates, univariable Cox analysis in the TARGET cohort identified prognosis-associated genes (Figure 2A). A least absolute shrinkage and selection operator (LASSO) Cox procedure determined the optimal penalty and yielded a parsimonious PTM-related prognostic model. The PTMS was defined as the weighted sum of the selected gene expression levels (Figures 2B,C). In the training cohort (TARGET), patients stratified by the median PTMS showed significantly poorer overall survival in the high-risk group, with satisfactory time-dependent discrimination (Figures 2D,E). Risk distribution plots indicated that deaths accumulated in the high-risk stratum, accompanied by a distinct expression pattern of model genes (Figure 2F). External validation in GSE21257 and GSE16091 reproduced the survival separation and predictive accuracy (Figures 2G–I). Multivariable Cox analysis confirmed that the PTMS was an independent prognostic factor after adjusting for clinical covariates (Figure 2J). Restricted cubic-spline analysis showed a monotonic increase in hazard with rising PTMS, and proportional-hazards diagnostics did not reveal material violations (Figures 2K,L). Decision-curve analyses demonstrated a higher net clinical benefit for PTMS, particularly when combined with clinical factors, across 1-, 3-, and 5-year horizons (Figures 2M–O). Calibration analysis indicated good agreement between predicted and observed survival probabilities (Figure 2P).

### PTMS-high denotes an immunosuppressed tumor ecosystem and inferior ICB benefit

**FIGURE 3:**
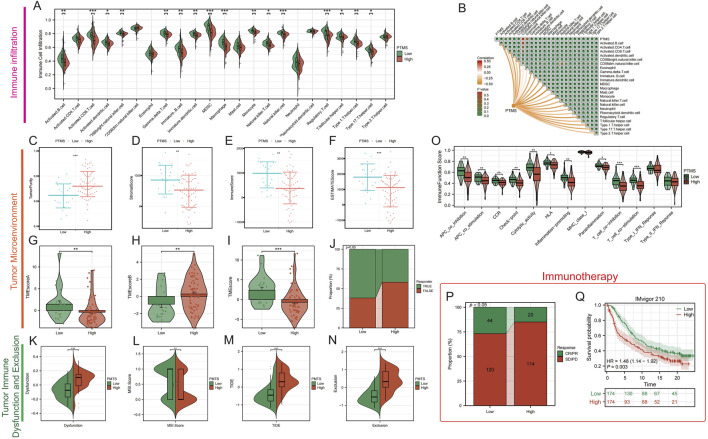
PTMS associates with an immunosuppressed TME and poorer ICB benefit. **(A)** Infiltration of immune cell types in PTMS-high vs. PTMS-low groups. **(B)** Spearman correlations between PTMS and immune cell abundances. **(C–F)** ESTIMATE metrics comparing groups. **(G–J)** Tumor microenvironment metrics between PTMS groups. **(K–N)** Tumor immune dysfunction/exclusion between PTMS groups. **(O)** Immune function signatures by PTMS group. **(P,Q)** IMvigor210 immunotherapy cohort: responder proportion by PTMS **(P)** and KM survival **(Q)**.

Across the TARGET cohort, ssGSEA indicated a globally immunosuppressed landscape in PTMS-high tumors compared with PTMS-low tumors (Figure 3A). Consistently, the PTMS value was negatively correlated with a majority of immune cells (Figure 3B). Tumor microenvironment (TME) metrics reinforced this pattern: PTMS-high tumors exhibited significantly lower ImmuneScore and ESTIMATE values with increased StromalScore, indicative of a less inflamed, more exclusionary TME (Figures 3C–F). Composite TME metrics (TMEscore-A/B and TMEscore) and an immune responder score further supported a suppressive microenvironment in PTMS-high tumors (Figures 3G–J). Measures of TIDE were increased, with higher TIDE, exclusion, and dysfunction scores, as well as differences in microsatellite instability (MSI), indicating enhanced immune evasion (Figures 3K–N). Immune function signatures (antigen-presenting cell (APC) co-stimulation, cytolytic activity, human leukocyte antigen (HLA), type I interferon (IFN) responses, and T-cell effector modules) were globally attenuated in PTMS-high tumors (Figure 3O). Consistently, in the IMvigor210 cohort, PTMS-high patients had fewer complete or partial clinical responses (CR/PR) and worse survival under PD-(L)1 blockade than PTMS-low patients (Figures 3P,Q). Reproducibility of the PTMS–immune infiltration pattern was confirmed in the GSE39058 validation dataset (Supplementary Figure S2).

### Model interpretation and single-cell localization of PTMS drivers

**FIGURE 4:**
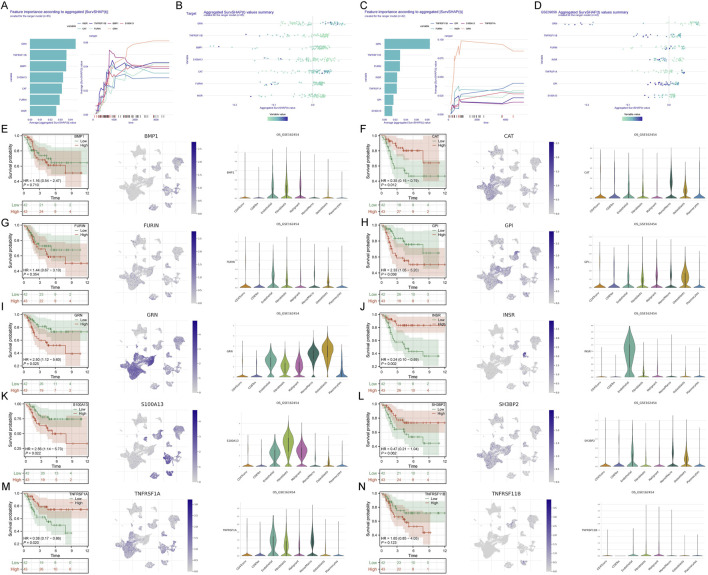
Interpretation of the PTM signature and single-cell localization of drivers. **(A,B)** TARGET: SHAP feature importance and beeswarm plots for genes contributing to the PTM score. **(C,D)** GSE21257 validation: SHAP importance and beeswarm plots using the TARGET-derived model. **(E–N)** For BMP1, CAT, FURIN, GPI, GRN, INSR, S100A13, SH3BP2, TNFRSF1A, and TNFRSF11B: KM overall survival (left), single-cell UMAP expression (middle), and cell-type violin plots (right).

To interpret the PTM-related prognostic model, we quantified feature contributions using SHAP in the training (TARGET) and validation (GSE21257) cohorts. In both datasets, a consistent set of genes dominated the risk score with stable importance across penalties, and sample-level effects were revealed by beeswarm plots (Figures 4A–D). Gene-level survival curves indicated that higher expression of most top contributors tracked with poorer overall survival, a pattern largely reproduced in the external cohort (Figures 4E–N). Single-cell mapping localized these drivers primarily to malignant and stromal compartments (osteoblast-/fibroblast-like clusters) with relatively low expression in lymphoid lineages. We next provide the rationale for focusing downstream analyses on GRN. Among the top contributors, GRN ranked prominently by SHAP, showed consistent adverse prognostic association across cohorts, and exhibited robust expression within malignant/stromal clusters in the single-cell atlas. Based on its model weight, prognostic relevance, and tumor/stroma-centered localization, we prioritized GRN as a key gene for subsequent analyses to elucidate its role in osteosarcoma.

### GRN associates with an immunosuppressive microenvironment

**FIGURE 5:**
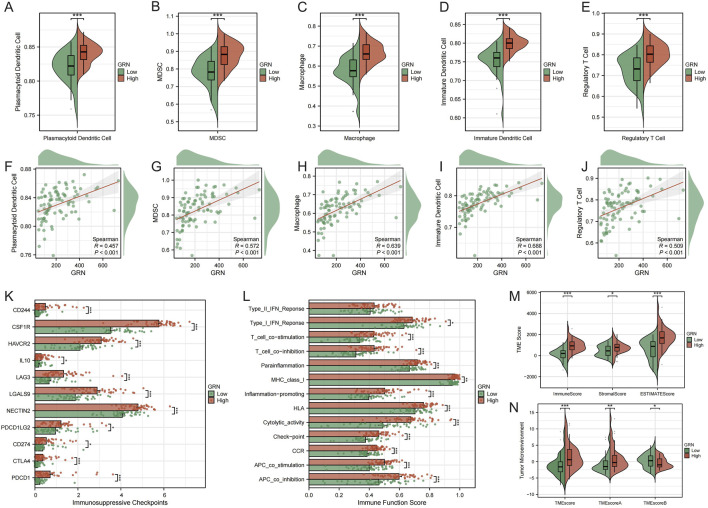
GRN links to an immunosuppressive TME. **(A–E)** Higher estimated infiltration of plasmacytoid dendritic cells, MDSCs, macrophages, immature dendritic cells, and Tregs in GRN-high tumors. **(F–J)** Positive correlations between GRN expression and the above cell populations. **(K)** Upregulated inhibitory checkpoints in GRN-high tumors. **(L)** Immune function modules: reduced cytolytic activity/APC co-stimulation with shifts toward T-cell inhibition and weakened IFN/HLA signals in GRN-high tumors. **(M,N)** Microenvironment scores: lower ImmuneScore/ESTIMATE and unfavorable TMEscore metrics in GRN-high tumors.

Stratification by GRN expression revealed higher infiltration of immunosuppressive/antigen-presenting lineages in the GRN-high group, including plasmacytoid dendritic cells, MDSCs, macrophages, immature dendritic cells, and regulatory T cells (Tregs) (Figures 5A–E). Consistently, GRN levels correlated positively with the abundance of these immunosuppressive cells (Figures 5F–J). Immunosuppressive checkpoint profiling showed broad upregulation of inhibitory receptors/ligands in GRN-high tumors, indicating intensified immune evasion signaling (Figure 5K). Immune function signatures further supported this pattern. GRN-high tumors displayed attenuated cytolytic activity and APC co-stimulation, with shifts toward T-cell inhibition and impaired IFN responses and HLA presentation (Figure 5L). Microenvironment scores separated the groups, with GRN-high cases showing lower ImmuneScore/ESTIMATE values and increased stromal content, as well as unfavorable TMEscore metrics (Figures 5M,N). Collectively, elevated GRN marks a TME that is quantitatively and qualitatively immunosuppressed in osteosarcoma.

### Drug sensitivity and molecular docking of GRN

**FIGURE 6:**
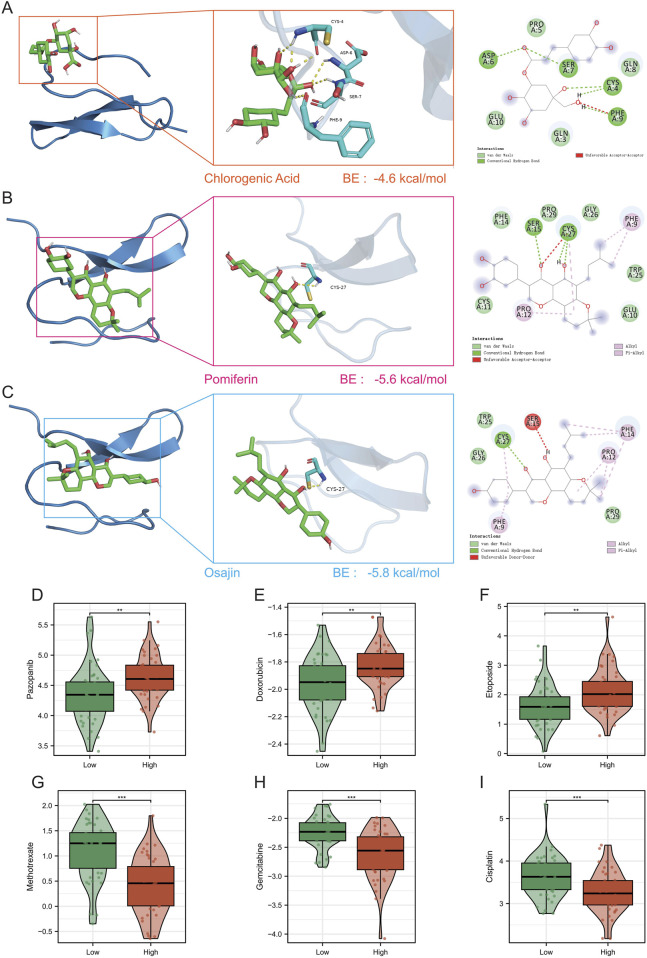
GRN docking and drug sensitivity. **(A–C)** Predicted binding of chlorogenic acid (A, −4.6 kcal/mol), pomiferin (B, −5.6 kcal/mol), and osajin (C, −5.8 kcal/mol) to the GRN pocket. **(D–I)** Predicted log_10_(IC_50_) by GRN status: paclitaxel **(D)**, docetaxel **(E)**, bortezomib **(F)**, methotrexate **(G)**, gemcitabine **(H)**, and cisplatin **(I)**.

Structure-based docking suggested that three natural products: chlorogenic acid, pomiferin, and osajin, fit a conserved pocket of GRN with favorable binding energies (−4.6 kcal/mol, −5.6 kcal/mol, and −5.8 kcal/mol, respectively), forming multiple hydrogen bonds and hydrophobic interactions consistent with stable complexes (Figures 6A–C). Drug–response analysis further connected GRN to therapy sensitivity: the GRN-high group showed higher predicted IC_50_ values (reduced sensitivity) for paclitaxel, docetaxel, bortezomib, gemcitabine, and cisplatin, whereas methotrexate displayed the opposite trend (lower IC_50_ in the GRN-high group) (Figures 6D–I). Together, these data nominate GRN as a potential druggable node and indicate GRN-linked chemoresistance patterns with a putative vulnerability to methotrexate.

### Functional validation of GRN in osteosarcoma cells

**FIGURE 7:**
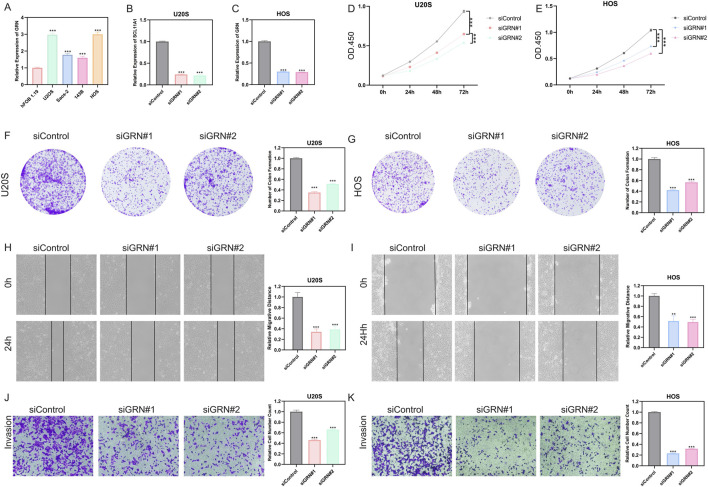
GRN drives osteosarcoma cell proliferation and motility. **(A)** GRN mRNA is elevated in OS cells (U2OS, 143B, and HOS) vs. hFOB1.19. **(B,C)** Efficient GRN knockdown by siGRN#1/#2 in U2OS **(B)** and HOS **(C)**. **(D,E)** CCK-8 assays show reduced growth after GRN silencing. **(F,G)** Fewer colonies upon GRN knockdown. **(H,I)** Wound-healing assays indicate impaired migration. **(J,K)** Transwell assays show decreased invasion.

qRT-PCR showed that GRN was significantly upregulated in U2OS, 143B, and HOS compared with hFOB1.19. Therefore, U2OS and HOS were selected for subsequent knockdown experiments (Figure 7A). We silenced GRN in U2OS and HOS cells using two independent siRNAs (siGRN#1/#2), both of which markedly reduced GRN mRNA levels compared with siControl (Figures 7B,C). GRN knockdown significantly impaired cell growth by CCK-8 assays across 24–72 h (Figures 7D,E) and decreased clonogenic capacity in both lines (Figures 7F,G). Wound-healing assays showed attenuated migratory ability upon GRN depletion (Figures 7H,I), and Matrigel Transwell assays demonstrated reduced invasive potential (Figures 7J,K). Collectively, GRN supports the proliferation, migration, and invasion of osteosarcoma cells *in vitro*.

## Discussion

OS remains a clinically challenging malignancy in which outcomes for patients with metastatic or relapsed disease have stagnated for decades (Corre et al., 2020). A deeper understanding of the molecular programs that shape tumor behavior and the tumor–immune ecosystem is urgently needed to guide risk stratification and treatment development (Cersosimo et al., 2020). PTM networks represent one such layer of regulation (Peng et al., 2022). By tuning protein stability, localization, and signaling flux, PTMs integrate oncogenic and microenvironmental cues that drive progression and therapeutic resistance (Jia et al., 2025; Miao et al., 2025). Yet PTM-related transcriptional programs have not been systematically connected to the single-cell architecture of OS or to clinically actionable phenotypes.

Mechanistically, dysregulated PTMs can reprogram immune ecology through several interconnected axes. Aberrant phosphorylation and ubiquitination influence the stability and trafficking of immune checkpoints and costimulatory receptors, thereby modulating antigen recognition and T-cell activation thresholds (Yu and Yao, 2024). Similarly, glycosylation of extracellular matrix and receptor proteins alters cell–cell communication and affects leukocyte infiltration. In osteosarcoma, where stromal dominance and matrix rigidity are defining features, PTM-driven remodeling of extracellular matrix (ECM) components and cytokine gradients likely contributes to T-cell exclusion and myeloid polarization.

We first delineated the single-cell landscape of OS to define cellular heterogeneity and identify PTM- and immune-related gene intersections. From the three-way intersection of tumor DEGs with PTM and immune repertoires, we derived a PTMS that was consistently prognostic across cohorts and independent of clinical factors. The PTMS was tightly linked to an immunosuppressed TME, reflected by reduced Immune and ESTIMATE scores, enrichment of myeloid-dominant and regulatory lineages, higher TIDE signals of dysfunction and exclusion, and attenuation of cytolytic, interferon, and antigen-presentation signatures. While the IMvigor210 cohort provides a reference framework for immune checkpoint blockade outcomes, it represents a distinct cancer type with different genomic and microenvironmental contexts. Therefore, cross-tumor extrapolation should be interpreted with caution, and the observed association between PTMS and immune nonresponse is hypothesis-generating. Future immunotherapy cohorts specific to osteosarcoma are required to establish predictive validity. These observations align with the view that immune evasion in OS is multifactorial and is often orchestrated by stromal–myeloid networks that blunt T-cell activity.

Our findings align with emerging models in which PTM wiring modulates OS immune ecology. Mechanistically, glycosylation can stabilize PD-L1 at the tumor surface and alter matrix/receptor interactions that impede T-cell trafficking; ubiquitination controls checkpoint turnover and antigen-processing nodes; and phosphorylation integrates oncogenic signaling with interferon/NF-κB programs that polarize myeloid cells. Recent reviews and experimental reports detail these circuits and provide a translational scaffold for PTM-targeted strategies to de-suppress myeloid niches and improve ICB responsiveness. In OS specifically, contemporary immune microenvironment maps (single-cell/spatial) converge on myeloid–stromal axes as dominant drivers of exclusion, precisely the axes our PTM-anchored signature and GRN-centered analyses implicate. Prioritizing PTM enzymes or substrates that gate checkpoint stability, NF-κB tone, and myeloid polarization may therefore offer rational combination partners for ICB or chemotherapy in OS. The reproducible associations between model-derived scores and immune cell infiltration are in line with recent studies demonstrating that transcriptional signatures often mirror underlying TME composition (Wei et al., 2023).

Model interpretation highlighted a compact set of PTM-linked drivers with coherent single-cell localization in malignant and stromal compartments. We prioritized GRN (progranulin) for deeper study based on its contribution to the model, its adverse prognostic association, and its tumor- or stroma-centered expression. Progranulin is a pleiotropic growth factor implicated in cell survival, invasion, and immune modulation, including crosstalk with myeloid lineages and cytokine circuits. In our data, high GRN expression tracked with increased infiltration of plasmacytoid dendritic cells, MDSCs, macrophages, and Tregs. In parallel, we observed upregulation of inhibitory checkpoints and depressed cytolytic or APC costimulatory programs, which together are consistent with an immunosuppressed and exclusionary TME. Functionally, GRN knockdown curtailed proliferation, clonogenicity, migration, and invasion of OS cells, supporting a tumor-promoting role.

We then focused the discussion on the biology of GRN in tumors. GRN encodes a secreted glycoprotein that is cleaved into granulin peptides and signals in an autocrine and paracrine manner to promote cell-cycle progression, survival, migration, and matrix remodeling (Saeedi-Boroujeni et al., 2023). In cancer settings, GRN engages PI3K–AKT, MAPK–ERK, and NF-κB pathways, thereby enhancing proliferation and stress resistance (Wang et al., 2021; Hu et al., 2012). At the immune–stromal interface, GRN modulates myeloid programs and cytokine networks. This modulation fosters macrophage recruitment and polarization toward immunoregulatory phenotypes, dampens cytotoxic T-cell activity, and supports a TGF-β-rich and stromalized niche that impedes lymphocyte trafficking (Zhang et al., 2024; Gan et al., 2024; Arechavaleta-Velasco et al., 2017). These mechanisms align with our findings that GRN-high osteosarcomas display enriched myeloid and Treg infiltration, elevated inhibitory checkpoints, and reduced cytolytic and APC-stimulatory functions. Together, they point to a tumor- or stroma-centric axis through which GRN drives immune suppression and malignant aggressiveness in OS.

Our study has limitations. The modeling relies on retrospective public cohorts with differences in platforms and clinical annotation. We validated across datasets and used established statistics for discrimination, calibration, and net clinical benefit, yet prospective testing is still required. PTMS is derived from transcriptomes and does not directly quantify protein-level PTMs. Future work that integrates phospho-, glyco-, and ubiquitin-proteomics may refine pathway mapping. Immune inference methods, such as ssGSEA, ESTIMATE, and TIDE, capture broad patterns but do not replace orthogonal measurements, such as multiplex immunohistochemistry or spatial transcriptomics. Predictions from docking and IC_50_ modeling are computational and need medicinal chemistry and pharmacology to be translated into therapies. Finally, the GRN axis likely acts within a broader network. Dissecting its interplay with myeloid signaling and matrix remodeling may reveal synergistic therapeutic strategies. Future work should integrate prospective osteosarcoma cohorts, apply spatial and proteomic immune profiling, and perform functional validation in animal or co-culture models to substantiate the mechanistic and therapeutic implications of the PTM–immune axis.

## Conclusion

In summary, linking PTM-anchored gene programs to single-cell architecture and immune ecology provides a framework to explain adverse prognosis and immune suppression in OS and indicates actionable biology. PTMS may complement current clinicopathologic factors for risk stratification, and GRN emerges as a tumor- or stroma-intrinsic node with therapeutic potential. Prospective validation combined with mechanistic and pharmacologic studies, ideally with spatial and proteomic profiling, will be essential to test PTM-targeted and GRN-directed interventions, alone or in combination with chemotherapy and immune checkpoint blockade.

## Data Availability

The datasets analyzed in this study are publicly available. Bulk transcriptomic and clinical data for osteosarcoma were obtained from the TARGET database and the Gene Expression Omnibus (GEO). Single-cell RNA-seq data (GSE162454) were accessed via the TISCH2 database.

## Supplementary material

SUPPLEMENTARY FIGURE S1 Overview of the study workflow.

SUPPLEMENTARY FIGURE S2 Validation of PTMS–immune associations in GSE39058. **(A)** ssGSEA-based immune cell infiltration heatmap. **(B)** Differences in stromal, immune, and ESTIMATE scores between PTMS strata. **(C)** TMEscore-A, TMEscore-B, and composite TMEscores in high and low PTMS groups. **(D,E)** T-cell dysfunction and exclusion scores, indicating impaired cytotoxic activity in PTMS-high tumors. **(F)** TIDE scores predicting reduced ICB responsiveness in high-PTMS samples. **(G)** MSI scores comparing the two subgroups.
