# Supplementary figures and images for: Single-cell dissection of PTM-related networks reveals an immunosuppressed osteosarcoma ecosystem
Source: Front Mol Biosci. 2025 Dec 18;12:1718941. doi: 10.3389/fmolb.2025.1718941 (PMC12756076; doi:10.3389/fmolb.2025.1718941)

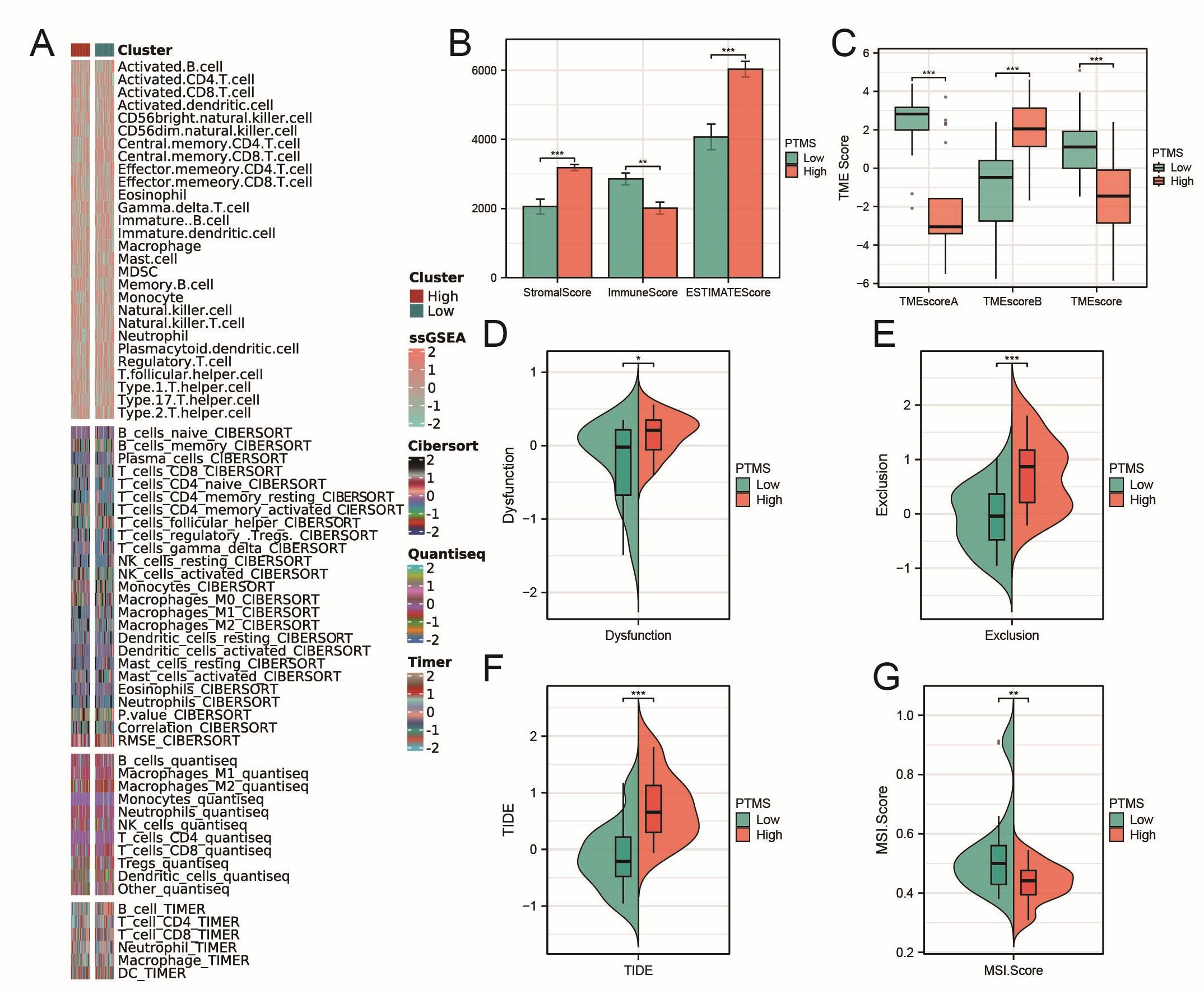

Supplement: Supplementary file 1 [file Image2.jpeg]

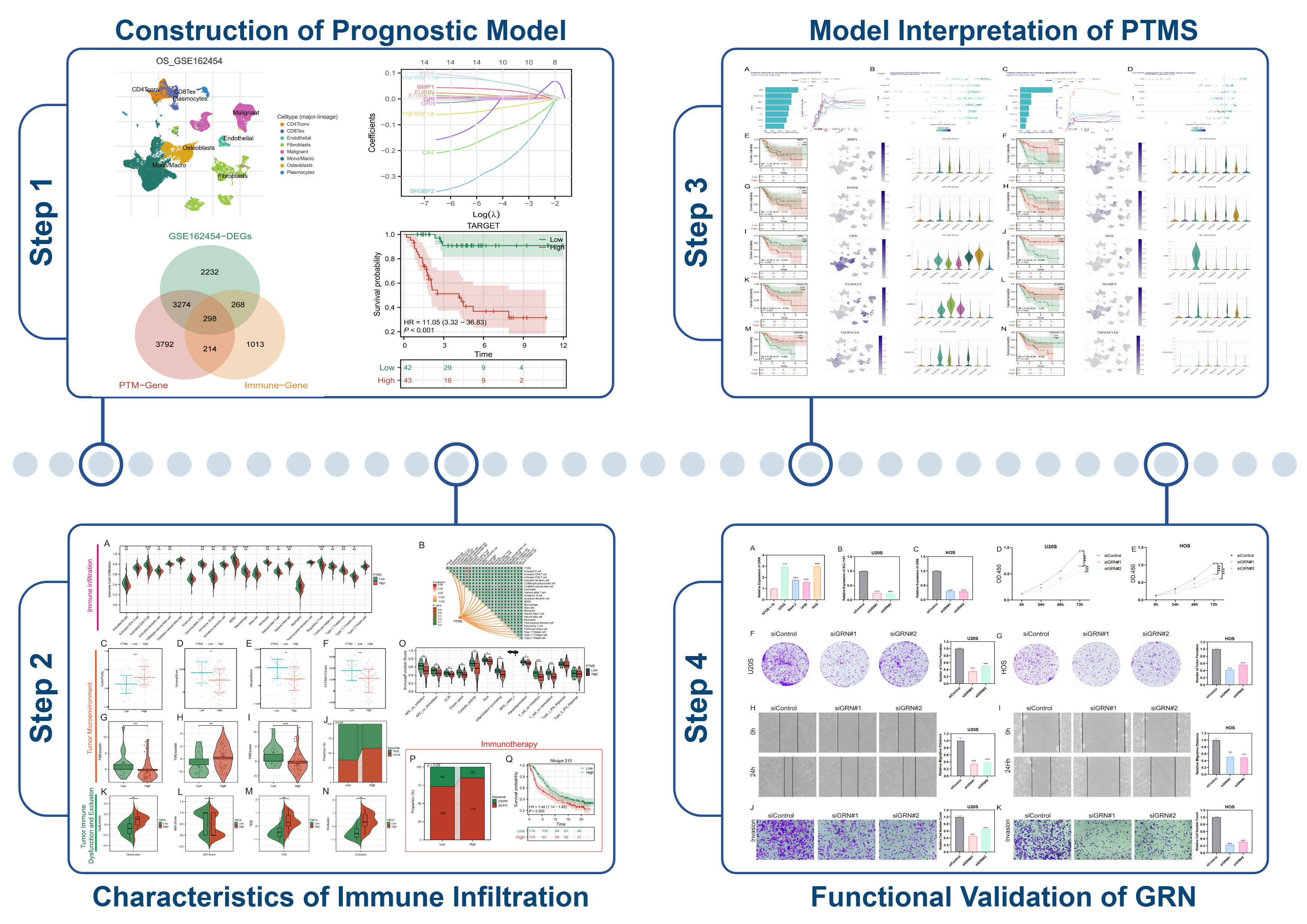

Supplement: Supplementary file 2 [file Image1.tif]
